# Anatomical Variations Encountered During the Posterior Approach to the Hip Joint

**DOI:** 10.3390/jfmk11040422

**Published:** 2026-10-15

**Authors:** Dimitrios Katsogiannis, George Triantafyllou, Christos Koutserimpas, Fotis Demetriou, Georgi P. Georgiev, George Tsakotos, Juan Jose Valenzuela-Fuenzalida, Juan Sanchis-Gimeno, Jose E. León-Rojas, Maria Piagkou

**Affiliations:** 1 Department of Anatomy, Faculty of Health Sciences, School of Medicine, National and Kapodistrian University of Athens, 11527 Athens, Greece; dimi.katsogiannis@gmail.com (D.K.); georgerose406@gmail.com (G.T.); fotisdemetriou2000@gmail.com (F.D.); gtsakotos@gmail.com (G.T.); mapian@med.uoa.gr (M.P.); 2 School of Health Rehabilitation Sciences, University of Patras, 26504 Patras, Greece; koutserimpas@upatras.gr; 3 Department of Orthopaedics and Traumatology, University Hospital Queen Giovanna—ISUL, Medical University of Sofia, 1431 Sofia, Bulgaria; georgievgp@yahoo.com; 4 Faculty of Medicine, Andrés Bello University, Santiago 8370146, Chile; juan.kine.2015@gmail.com; 5 Department of Chemical and Biological Sciences, Faculty of Health Sciences, Bernardo O’Higgins University, Santiago 8370993, Chile; 6 GIAVAL Research Group, Department of Anatomy and Human Embryology, Faculty of Medicine, University of Valencia, 46001 Valencia, Spain; juan.sanchis@uv.es; 7 Grupo de Investigación Bienestar, Salud y Sociedad, Escuela de Psicología y Educación, Universidad de Las América, Quito 170124, Ecuador

**Keywords:** posterior hip approach, total hip arthroplasty, variations, anatomy

## Abstract

The posterior approach to the hip joint remains widely used for primary total hip arthroplasty because it provides extensive exposure while preserving the abductor mechanism. Safe execution, however, depends on recognizing anatomical variation within the gluteal region. This narrative review synthesizes variations encountered sequentially during posterior hip exposure and organizes them into superficial, muscular, neurovascular, and osseous domains. Key considerations include branching patterns of the posterior femoral cutaneous and superior cluneal nerves, morphological variation in the short external rotators and quadratus femoris, variation in the sciatic nerve-piriformis relationship, persistent embryonic vessels, os acetabuli, and proximal femoral morphology. The evidence base is heterogeneous and is dominated by cadaveric and anatomical studies, with several prevalence estimates derived from small series. Accordingly, reported frequencies should be interpreted in light of study design and sample size. By arranging these findings according to surgical planes and distinguishing documented clinical complications from anatomy-based theoretical risks, this review provides a practical anatomical roadmap for preoperative planning and intraoperative orientation. The direct effect of many variants on surgical outcomes remains uncertain and warrants prospective clinical study.

## 1. Introduction

The hip region demonstrates complex interactions between muscular, capsular, and neurovascular structures, with anatomical variability influencing both stability and surgical exposure [1].

The posterior approach to the hip joint remains the most common surgical route utilized for primary total hip arthroplasty [2,3,4]. Its widespread clinical adoption is rooted in its versatility and ability to provide extensive exposure of the acetabulum and femur, which is essential for both routine reconstructions and the management of complex bone defects or fractures [2,4]. Although it is characterized as a muscle-splitting technique that requires the blunt division of the gluteus maximus and the detachment of the short external rotators, it offers the distinct advantage of sparing the abductor muscles during surgical exposure [3]. However, the safety and reliability of this approach depend heavily on the surgeon’s ability to navigate deep gluteal landmarks that are subject to significant anatomical variation.

Unexpected morphological variants can complicate the surgical dissection even for experienced surgeons, potentially leading to iatrogenic lesions with neural [5] and arterial injuries [6]. This is particularly critical for surgeons who rely on textbook landmarks for orientation, which may not account for the high prevalence of accessory structures or shifted neurovascular pathways.

Historically, anatomical reviews relevant to hip arthroplasty have remained highly compartmentalized, focusing on isolated domains such as sciatic nerve anomalies [7]. Consequently, a comprehensive synthesis that maps these variations chronologically to the sequential steps of the posterior approach is currently lacking. Therefore, the aim of this paper is to provide a synthesis of the anatomical variants—superficial, muscular, neurovascular, and osseous—encountered from skin incision to joint exposure. By synthesizing current prevalence data and clinical implications, we aim to provide surgeons with an essential “anatomical map” to enhance surgical safety and prevent avoidable complications.

## 2. Literature Search Strategy

This narrative review was performed according to the Scale for the Assessment of Narrative Review Articles (SANRA) guidelines. A targeted literature search was performed in MEDLINE (PubMed), Scopus and Google Scholar until May 2026. The search strategy utilized Boolean operators and combinations of the following keywords and Medical Subject Headings (MeSH) terms: arthroplasty, replacement, hip, total hip arthroplasty, posterior approach, anatomical variations, sciatic nerve, piriformis muscle, short external rotators, quadratus femoris, posterior femoral cutaneous nerve, superior cluneal nerve, inferior gluteal artery, and os acetabuli.

Inclusion criteria comprised cadaveric anatomical studies, prospective clinical studies, case reports, systematic reviews, and meta-analyses published in English that detailed morphological variations relevant to the posterior surgical exposure of the hip joint. A risk-of-bias assessment tool was not applied given the narrative nature of this review and the heterogeneity of the source material (ranging from single case reports to meta-analyses); studies were critically appraised for sample size and methodological quality.

## 3. Variations in Superficial Dissection

### 3.1. Posterior Femoral Cutaneous Nerve

The posterior femoral cutaneous nerve of the thigh (PFCN) arises from the posterior divisions of the anterior rami of the S1 and S2 nerves and the anterior divisions of the anterior rami of nerves S2 and S3. The nerve first appears lateral to the anterior sacral foramina, positioned underneath the psoas parietal pelvic fascia. It exits the pelvis inferior to the piriformis muscle (PM) via the greater sciatic foramen. The main nerve branches supply the cutaneous distribution of the posterior thigh, the skin over the medial thigh, the popliteal fossa and sometimes the proximal aspect of the posterior leg [8].

The PFCN demonstrates highly reproducible topographic relationships within the posterior gluteal region. During posterior exposure, the nerve is consistently located superficial to the sciatic nerve, lying 30.5 mm lateral to the central tip of the ischial tuberosity. The branches of the PFCN cross the hamstring tendons at predictable proximal and distal levels [9]. The PFCN was closest to the inferior border of the gluteus maximus at 45.8 mm (range: 13.6–62.1 mm) lateral to the ischial tuberosity. Most importantly, the PFCN maintained a consistent lateral position relative to the central tip of the ischial tuberosity, remaining in direct contact with the sciatic nerve in all cases [9].

The identification of muscular branches from the PFCN or its perineal division supplying the gluteus maximus has direct implications during the posterior approach to the hip. Detachment and retraction of the gluteus maximus-particularly when splitting the muscle along its fibers-may inadvertently compromise these variant motor contributions [10]. Injury may lead to localized sensory loss or dysesthesia of the posterior thigh and perineal regions and potentially diminish gluteus maximus function, subtly affecting postoperative gait mechanics [10]. Knowledge of predictable distances between the PFCN and the ischial tuberosity is essential when placing distal retractors or performing capsular work near the quadratus femoris [9]. Crossing branches over hamstring tendons may be injured during overzealous medial retraction, leading to perineal dysesthesia or neuropathic pain. During posterior approach or capsular work, retractors placed medial to the hamstring origin risk compressing transverse perineal branches [9].

### 3.2. Superior Cluneal Nerve

In addition to the PFCN, the superior cluneal nerve (SCN) represents another superficial neural structure of relevance in posterior hip approach surgery. Originating from the dorsal rami of the L1–L3 spinal nerves, the SCN frequently demonstrates a medial branch that traverses osteofibrous tunnels along the iliac crest and maintains a consistent relationship with the posterior superior iliac spine, with mean distances of 45.2 ± 11.2 mm and 65.3 ± 8.2 mm from the midline [11].

Kuniya et al. [12] reported that in 39% of cases, the medial branch of the SCN traverses osteofibrous tunnels along the iliac crest. The SCN variable course—especially the tendency of its medial branch to traverse osteofibrous tunnels near the iliac crest—poses a risk during placement of posterior retractors or during fascial elevation and palpation over the crest when establishing the surgical interval [11].

Entrapment or iatrogenic injury to the SCN may manifest postoperatively as neuropathic pain, potentially misinterpreted as deep gluteal syndrome or referred hip pain [11]. Awareness of tunnel-bound SCN branches is essential when preparing the patient for posterior arthroplasty, as traction or compression at the crest may trigger chronic postoperative dysesthesia or postoperative gluteal numbness or burning pain [11].

## 4. Muscular Variations in Deep Dissection

### 4.1. Piriformis Muscle

The PM is the key anatomical landmark in the posterior approach for total hip arthroplasty. Although different variations in the PM in relation to the SN are well known, the position and variation in its insertion on the proximal femur are scarcely described [13]. Roche et al. [13] reported that the piriformis tendon typically inserts into the crest of the greater trochanter; extensions to the fibrous capsule of the hip joint are also described.

Georgiadis et al. [14] reported that the piriformis tendon is inserted relatively high on the greater trochanter, with a mean distance of around 2.2 cm anterior to its posterior border and located anterior to the mid-axis of the femoral canal. Roche et al. [13] considered that the PM could be damaged with particular instruments during posterior approaches for total hip replacement.

### 4.2. Short External Rotators

While traditionally regarded in classical anatomy as having a constant appearance and serving as standard landmarks in posterior approaches to the hip, the short external rotator muscles (gemelli and obturator internus) actually exhibit notable morphological variability [15] (Figure 1).

Aung et al. [16] investigated 39 cadaveric limbs and described that muscular fibers of the obturator internus (OI) emerge within the muscle and are evident from the lateral surface of the pelvic floor. In most of the cases, the gemelli were divided into superior and inferior parts, but in two specimens, an extension of the OI fibers continued to join the inferior gemellus (IG), while in some specimens the boundary between the two could not be clearly distinguished [16].

Classical anatomical literature has long recognized that gemelli may be absent or fused with the OI [17]. Shinohara [18] studied 14 specimens and observed that in 5 limbs (36%) the superior gemellus (SG) inserts into the superior margin of the OI tendon and the IG terminates on the inferior margin of the same tendon [18]. This type of anatomical variability produces a configuration in which the tendon appears “sandwiched” between the two gemelli and all three muscles lie on the same plane. On the other hand, the remaining 9 specimens (64%) had the gemelli muscle covering the superficial surface of the OI tendon [18]. Furthermore, Arifoglu et al. [19] described a cadaveric case with an additional SG, found inferiorly to the tibial nerve and the lower part of the PM. Lastly, in a recent cadaveric study of 60 lower limbs, the SG was found to be absent in 13.3% of cases, while the IG was absent in 20% [20]. When present, the SG exhibited a double-bellied configuration in 10% of cases (with the upper belly fused to the piriformis) and presented as a single belly fused to the piriformis tendon in 11.7% of cases. The IG demonstrated a double-bellied configuration in 5% of specimens [20].

### 4.3. Obturator Externus Muscle

The obturator externus (OE) is a triangular bipennate muscle originating from the obturator foramen and inserting via an intramuscular tendon beneath the acetabulum [8].

Assi et al. [21] described the obturator externus (OE) as a triangular bipennate structure that originates from the obturator foramen and inserts via an intramuscular tendon beneath the acetabulum. In five of eighteen cases (27.8%), the muscle exhibited two distinct heads—cranial and caudal—that merged into the distal tendon [21]. Variant dual-headed OE morphology and orthogonally oriented hip triceps musculature may influence deep exposure during detachment or tagging of short external rotators [21]. A bifid OE may obscure the distal tendon footprint, causing misidentification of the true capsular plane [21]. From an anatomical perspective, it is hypothesized that the exaggerated shortening capacity of a variant OE could theoretically contribute to postoperative limitations in external rotation if inadvertently tethered [21]. Recognizing this consistent relationship facilitates surgical identification during the posterior hip approach, especially when reattaching short external rotator muscles, helping surgeons orient themselves even when the PM morphology is atypical [21].

### 4.4. Quadratus Femoris Muscle

The quadratus femoris (QF) muscle has distinct anatomical variability [22]. Based on the number of muscle heads and their insertional configuration, Olewnik et al. [22] categorized quadratus femoris into three morphological types (Figure 2):

- Type I—a single muscle extending from the lateral border of the ischial tuberosity to the intertrochanteric crest.
- Type II—a double quadratus femoris muscle with superior and inferior components inserting separately along the intertrochanteric crest.
- Type III—tripartite morphology consisting of three distinct muscular portions arising from the ischial tuberosity, inserting correspondingly on the posterior femur.

**Figure 2:**
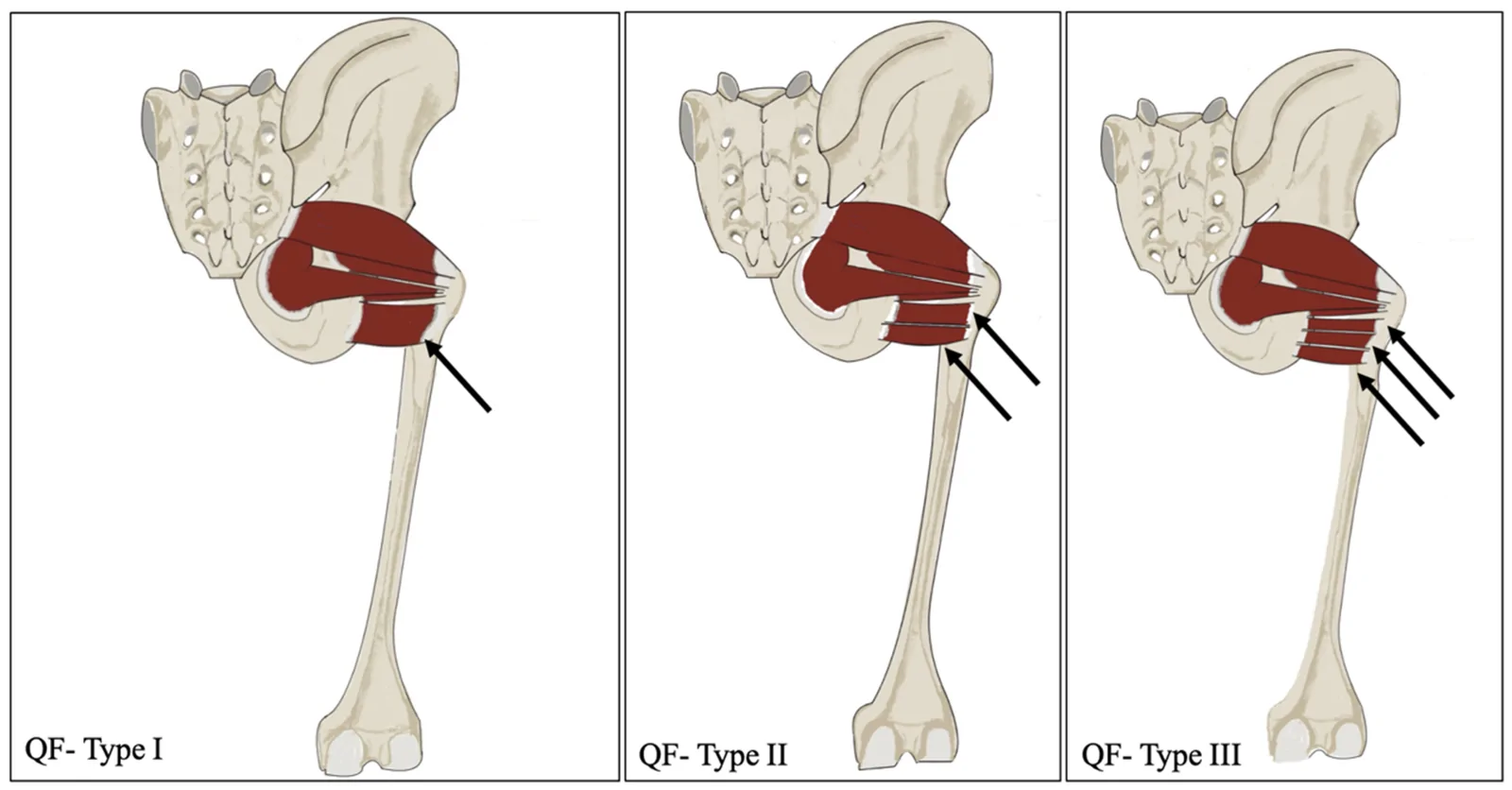
The variations in the quadratus femoris (QF) as described by Olewnik et al. (2024) [22]. Image Credit: Mr. Fotis Demetriou.

Multiple-bellied QF morphology may broaden the muscular mass occupying the space between the sciatic nerve and the femur. During femoral preparation, excessive medial retraction may compress these variant portions against the sciatic nerve [22]. Furthermore, due to its spatial arrangement, aberrant QF morphology could theoretically distort the visual outline of the inferior capsular recess, introducing a potential surgical risk for incomplete capsulotomy or inadvertent violation of the tendon [22].

### 4.5. Tendon Patterns

Nevertheless, the spatial arrangement of the PM, the OI tendons and the posterior hip capsule forms a consistent anatomical complex during posterior hip exposure. Pine et al. [23] studied 29 cadaveric hips and categorized the most common configuration into four different types:

- Type I (55%): independent horizontal and vertical tendon orientation.
- Type II (17%): oblique convergence of tendons (<90°).
- Type III (17%): piriformis tendon located directly above obturator internus.
- Type IV (10%): fused tendons forming a broad common insertion.

Variability in the orientation, convergence angle, or fusion of the PM and OI tendons has direct surgical relevance, as the posterior approach relies on identifying and releasing the short external rotators in a stepwise manner [23,24]. Fused or obliquely convergent tendon morphologies may blur the distinction between the PM and conjoined tendon, increasing the risk of incomplete or excessive release. In the posterior approach, where posterior soft-tissue tension contributes to dislocation resistance, a fused tendon structure can compromise postoperative stability by undermining appropriate reattachment [23,24].

## 5. Neurovascular Variations in Deep Dissection

### 5.1. Inferior Gluteal Nerve

The inferior gluteal nerve (IGN) usually arises from the dorsal part of the L5, and the S1 and S2 ventral rami of the sacral plexus. It leaves the pelvis through the greater sciatic foramen, caudally to the PM and divides into branches to innervate the gluteus maximus (GM) muscle [25].

Tempski et al. [25] reported seven variants of IGN in regard to the PM. IGN, especially its variations, is at risk of iatrogenic injury during posterior and posterolateral surgical approaches to the hip [25,26,27,28]. The posterior approach is widely used due to the excellent exposure of the femoral shaft, particularly in revision arthroplasty [28].

The vulnerability of the IGN is determined by its close anatomical relationship with the deep surface of the GM, and the nerve is at risk when this muscle is split more than 5 cm proximal to the greater trochanter [26]. Apaydin et al. [27] described a triangular anatomical region defined by the posterior inferior iliac spine, ischial tuberosity, and greater trochanter, with its superior portion representing a “danger zone” containing the IGN and its branches.

### 5.2. Inferior Gluteal Artery

The inferior gluteal artery (IGA) arises within the pelvis as a branch of the anterior trunk of the internal iliac artery. It progresses inferiorly between the S2 and S3 nerve roots and exits the pelvic cavity through the greater sciatic foramen. After that, the IGA, accompanied by the inferior gluteal nerve, enters the gluteal region inferiorly to the PM and proceeds downwards between the ischial tuberosity and the greater trochanter [8]. There it continues under the gluteus maximus in the fascia of the biceps femoris and semitendinosus muscles, where it gives a small direct cutaneous branch that accompanies the PFCN to the popliteal fossa [8]. An IGA passing through the PM places the vessel at particular risk during piriformis detachment or when placing superior retractors. Bleeding from this variant artery may be brisk and difficult to control due to its deep trajectory [29].

### 5.3. Persistent Embryonic Vessels

Persistence of the primitive embryonic vessels of the lower limb, known as the sciatic artery or vein, is an important neurovascular variant that can be implicated during the posterior hip approach. The sciatic artery normally involutes in the third month of embryonic development, but it persists in 0.03–0.06% of the population [30]. In these cases, the sciatic artery emerges from the internal iliac artery and travels through the greater sciatic foramen in close relationship with the sciatic nerve. On the other hand, a persistent sciatic vein is also a rare variant, but it has a high prevalence in patients with Klippel–Trenaunay syndrome [31]. Piagkou et al. [32] reported a cadaveric case with bilateral and symmetrical persistent sciatic veins that initiated from the popliteal vein, traveled through the abductor magnus muscle, and drained into the common femoral vein. Persistent sciatic vessel segments or incomplete regression patterns increase the likelihood of encountering large-caliber vessels posterior to the hip joint or near the sciatic nerve. These vessels may be mistaken, but their injury can produce catastrophic bleeding [33] (Figure 3).

### 5.4. Sciatic Nerve

The sciatic nerve (SN), originating from L4-S3, exits the pelvis through the infrapiriform foramen and travels the posterior thigh before it divides into the tibial nerve (TN) and common peroneal nerve (CPN) [8]. Several anatomic variations in the relationship between the SN and the PM exist, initially described by Beaton and Anson [34] and used in the meta-analysis by Tomaszewski et al. [7], which resulted in the current classification system comprising seven variants (Figure 4):

- Type A—undivided SN passing below the PM.
- Type B—one division of the SN passing through, and the other below, the PM.
- Type C—one division above and the other below the PM.
- Type D—undivided SN passing through the PM.
- Type E—one division passing through and the other above the PM.
- Type F—undivided SN passing above the PM.
- Type G—divided SN passing below the PM.

**Figure 4:**
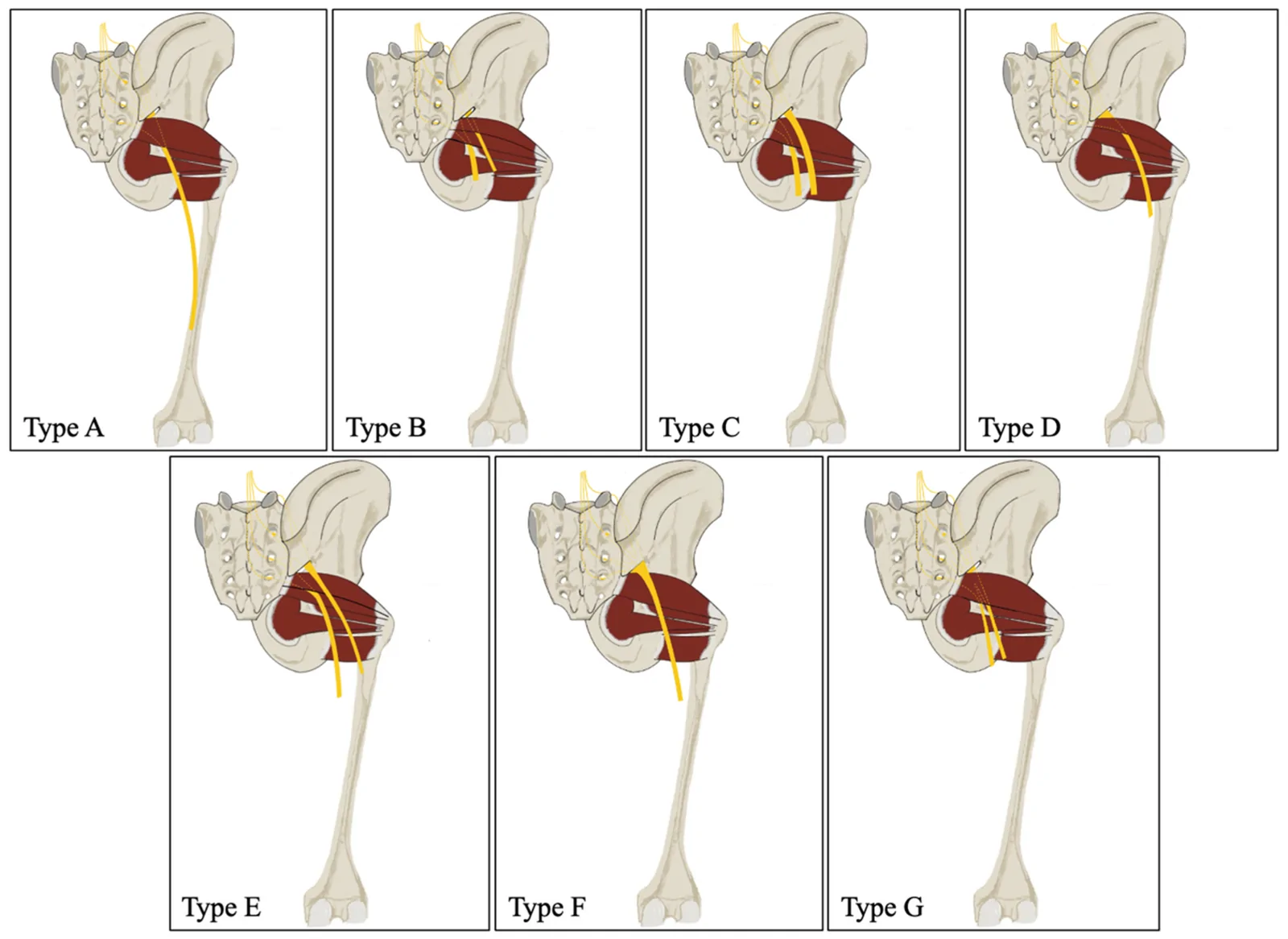
The types of sciatic nerve (SN)—piriformis muscle (PM) relationship. Image Credit: Mr. Fotis Demetriou.

Poutoglidou et al. [35] proceeded to the most recent systematic review and metanalysis involving 8257 samples and synthesized global data on these anatomical variants. Type A was the typical morphology with an estimated pooled prevalence of 90%. Total variants demonstrated a pooled prevalence of 13%. Specifically, Type B was the most frequent variant with a pooled prevalence of 8%. Types C and D were rare configurations with 2% and 1%, respectively. Subgroup analysis revealed marked geographical variation, with East Asian populations showing a significantly higher prevalence of Type B variants (24%) compared with Europe (9%), the USA (4%), and Africa (3%). A sex-related trend was also noted, as female specimens exhibited a higher prevalence of variants (13%) than males (6%) [35]. The predominance of the typical type A configuration—where the undivided sciatic nerve passes below the piriformis—helps establish predictable expectations during deep posterior exposure in hip arthroplasty [7,36]. However, the presence of less common variants, including divided sciatic nerves, duplicated piriformis bellies, or nerve branches traversing between piriformis slips, increases the risk of iatrogenic injury during piriformis release, external rotator detachment, or placement of deep retractors [7,36]. In type D or type E configurations, where the sciatic nerve or its divisions pierce the piriformis, even minor traction may precipitate neurapraxia. These variants also reduce the margin of safety when sweeping soft tissue off the posterior capsule, requiring surgeons to mobilize the piriformis with meticulous attention to avoid exposing or stretching an unexpectedly positioned nerve [7,36].

## 6. Osseous Variations in Deep Dissection

### 6.1. Accessory Ossicles

Os acetabuli, defined as unfused secondary ossification centers of the acetabular rim, constitute a distinct spectrum of acetabular rim ossifications that may appear as discrete ossicles along the acetabular margin and must be differentiated from fracture fragments or degenerative debris [37]. Their presence may be related to developmental anomalies, residual ossification centers, or chronic mechanical stress at the acetabular floor [37]. Yoshitani et al. [38] analyzed 107 screened articles and evaluated 8836 patients, among whom 604 were diagnosed with os acetabuli, confirming an overall prevalence range of 3.4–7.7%. Mistaking accessory nuclei for fracture fragments or osteophytes may lead to unnecessary debridement, risking capsular destabilization or inadvertent labral injury. These ossicles may affect the engagement of the acetabular component if not recognized on preoperative imaging, especially in dysplastic hips [37,38] (Figure 5).

### 6.2. Femoral Morphology

Proximal femoral osseous morphology has been investigated with particular attention to the relationship between femoral neck version, lesser trochanteric version, and the iliopsoas notch [39]. A strong positive correlation was identified between femoral neck and lesser trochanteric versions, indicating that the position of the lesser trochanter depends largely on femoral neck orientation and can serve as a surrogate marker of native anteversion [39]. Ollivier et al. [40] observed that proximal femoral geometry is influenced by patient morphotype and underlying pathology, suggesting that both body habitus and disease etiology systematically shape proximal femoral anatomy, with potential implications in hip arthroplasty planning.

Variations in native femoral neck version and femoral offset directly influence stability in the posterior approach, where posterior capsular and external rotator repair is critical. To mitigate dislocation risks, surgeons must account for the concept of combined anteversion—the sum of acetabular and femoral anteversion—ensuring the combined angle falls within a safe functional zone [41,42]. Hips with abnormal native anteversion require compensatory adjustment of the acetabular cup to achieve optimal combined anteversion and prevent posterior translation. Furthermore, restoring or increasing femoral offset is essential to optimize soft-tissue tension. Reduced femoral offset decreases the tension of the repaired posterior capsule and short external rotators, significantly increasing the risk of posterior instability [41,42].

## 7. Clinical and Surgical Implications

While some variations discussed in this review are linked to directly documented clinical complications (such as persistent sciatic vessels causing hemorrhage), other implications regarding soft-tissue balancing and retractor impingement represent theoretical risks extrapolated from cadaveric topography. Nonetheless, these anatomical insights remain critical for surgical safety and complication prevention during posterior hip arthroplasty. Large clinical series and registry analyses describe aggregate outcomes associated with the posterior approach but generally do not isolate the independent effect of individual anatomical variants [43,44,45,46,47]. Preoperative imaging may nevertheless identify acetabular rim ossicles and define proximal femoral morphology relevant to component planning [37,38,39,40,41,42].

During superficial dissection, awareness of the PFCN position 30.5 mm lateral to the ischial tuberosity [9] and the SCN osteofibrous tunnel course (39% prevalence) [11] is essential when placing retractors, as injury may cause chronic neuropathic pain mimicking deep gluteal syndrome [12].

Deep dissection requires heightened vigilance in the danger zone 32–55 mm posterior to the greater trochanter, particularly during the hip flexion beyond 30° with internal rotation, which reduces SN-to-femoral neck distance from 43.1 mm to 19.1 mm [44]. Kanawati et al. [45] also established that the SN is at risk during posterior approach after progressive hip flexion. These authors measured that at 0° of hip flexion with 90° internal rotation, the mean distance between the sciatic nerve and the femoral neck was 43.1 mm; the distance decreased with increasing hip flexion, reaching 36.1 mm, 28.8 mm, and 19.1 mm at 30°, 60°, and 90° of flexion, respectively. Moreover, in two cases, direct contact between the SN and the femoral neck was observed at 90° of flexion [45]. Recognition of SN-PM variants (total variants 13%, with Type B higher in females and East Asians) mandates careful piriformis mobilization, especially in Type D and E configurations where nerve divisions pierce the muscle [3]. Variant short external rotator morphology, including fused PM-OI tendons (reported as 10% in a 29-specimen study) [23], bifid OE (observed in 27.8% of an 18-specimen series) [21], and multipartite QF, may obscure capsular planes and increase risk of incomplete release or sciatic nerve compression [46].

Vascular awareness is also critical, as persistent sciatic vessels (0.03–0.06%) and variant inferior gluteal artery courses pose catastrophic bleeding risk during muscle detachment [47]. Finally, morphotype-specific variations in femoral anteversion and offset directly influence posterior soft-tissue tension; hips with increased anteversion require compensatory acetabular positioning to mitigate posterior dislocation risk [43]. These anatomical considerations underscore the necessity for individualized surgical planning, meticulous landmark-based dissection, and judicious retractor placement (Table 1).

## 8. Conclusions

Anatomical variability is encountered throughout posterior hip exposure, from superficial cutaneous nerves to the short external rotators, sciatic neurovascular relationships, and osseous morphology. The evidence base is predominantly cadaveric and anatomical, and several prevalence estimates are derived from small series. Accordingly, use this review as a topographic planning and awareness framework rather than as evidence that individual variants independently worsen surgical outcomes. When indicated, preoperative imaging, careful landmark-based dissection, and recognition of atypical anatomy may help surgeons anticipate potential pitfalls. Prospective clinical studies are needed to quantify the variant-specific risks of intraoperative complications, instability, and postoperative functional outcomes.

## Figures and Tables

**Figure 1:**
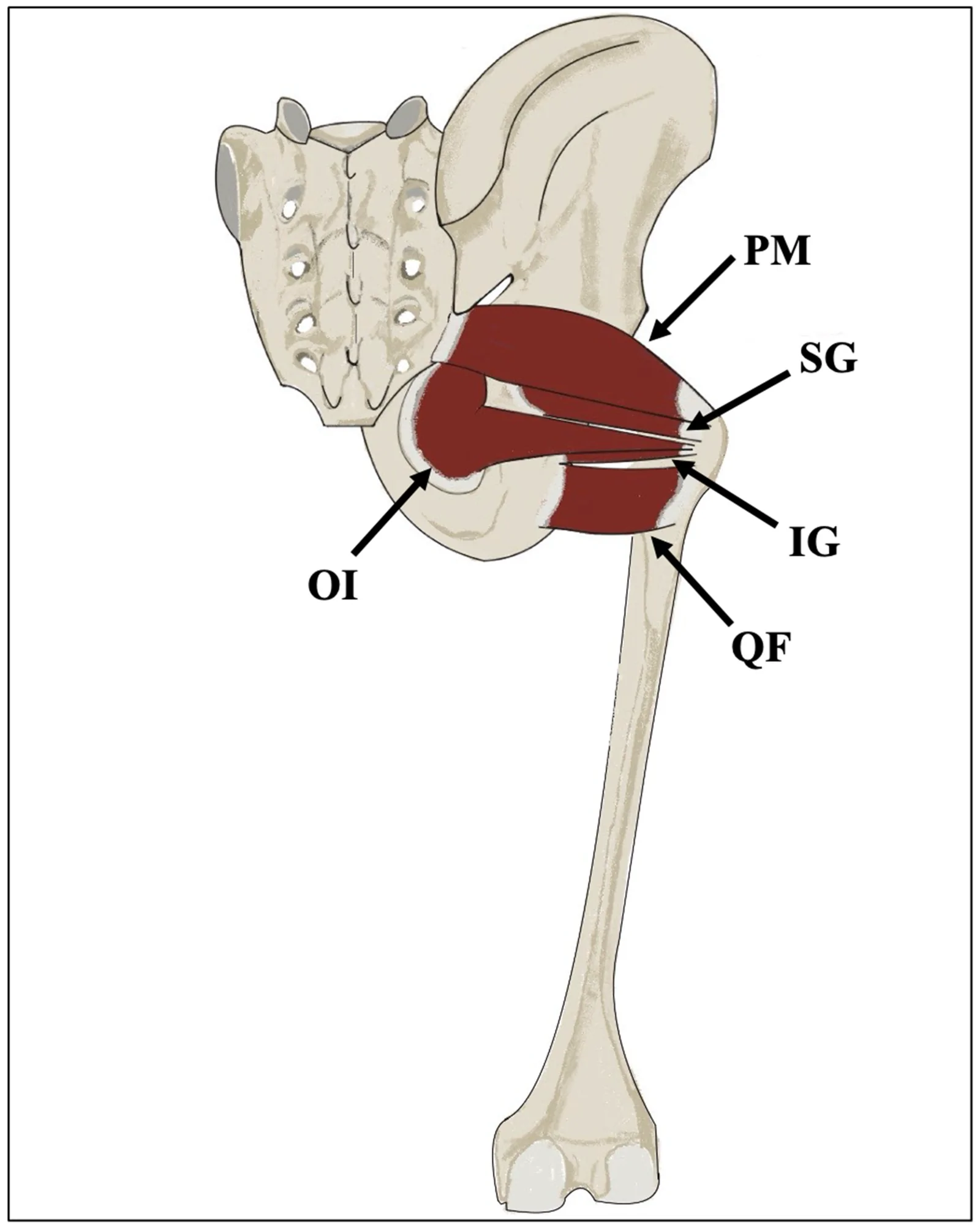
The typical musculature observed during posterior hip approach. PM—piriformis muscle, OI—obturator internus, SG—superior gemellus, IG—inferior gemellus, QF—quadratus femoris. Image Credit: Mr. Fotis Demetriou.

**Figure 3:**
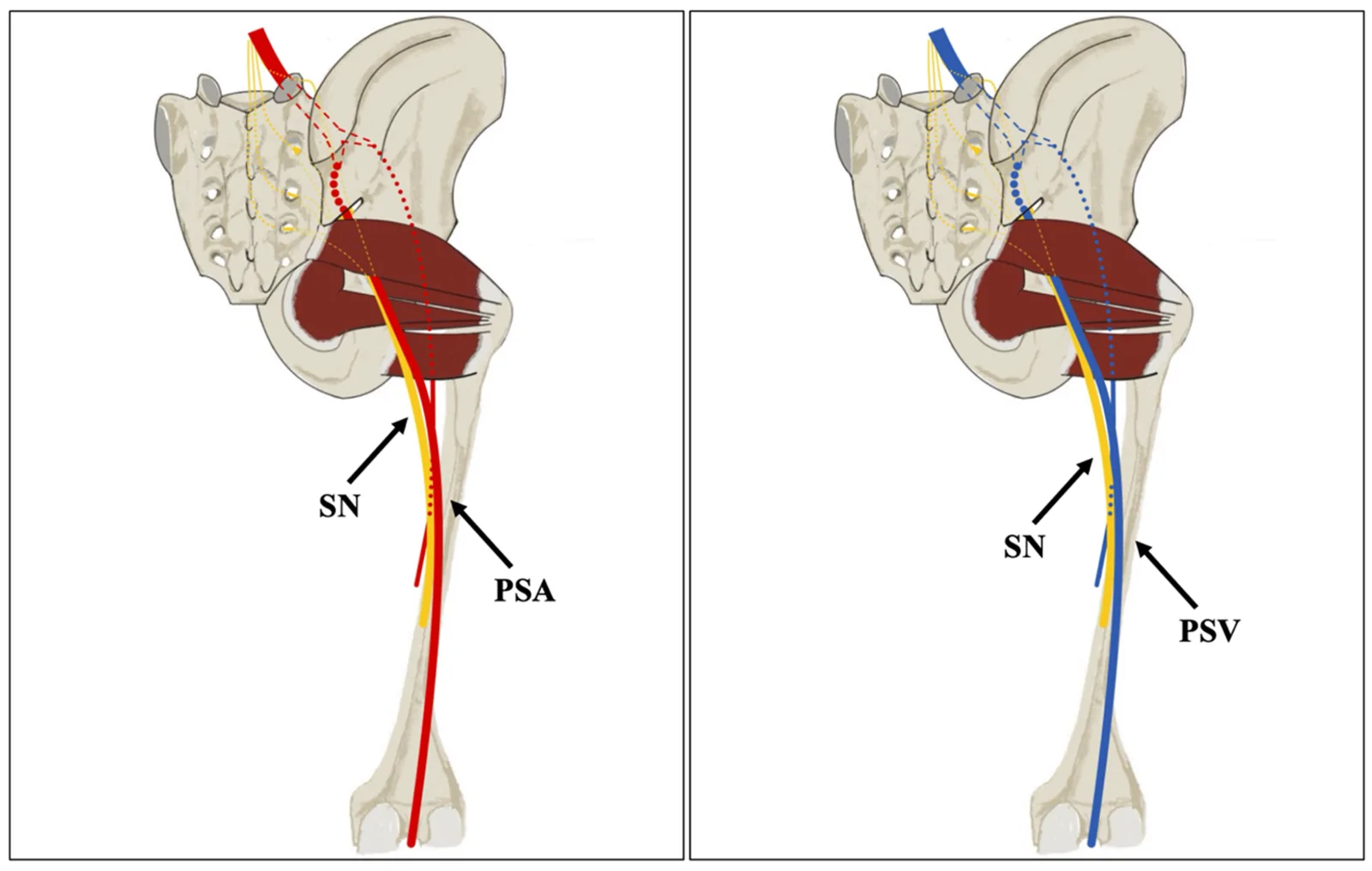
The persistent sciatic artery (PSA) and vein (PSV) variants that can be identified on the posterior thigh. Image Credit: Mr. Fotis Demetriou.

**Figure 5:**
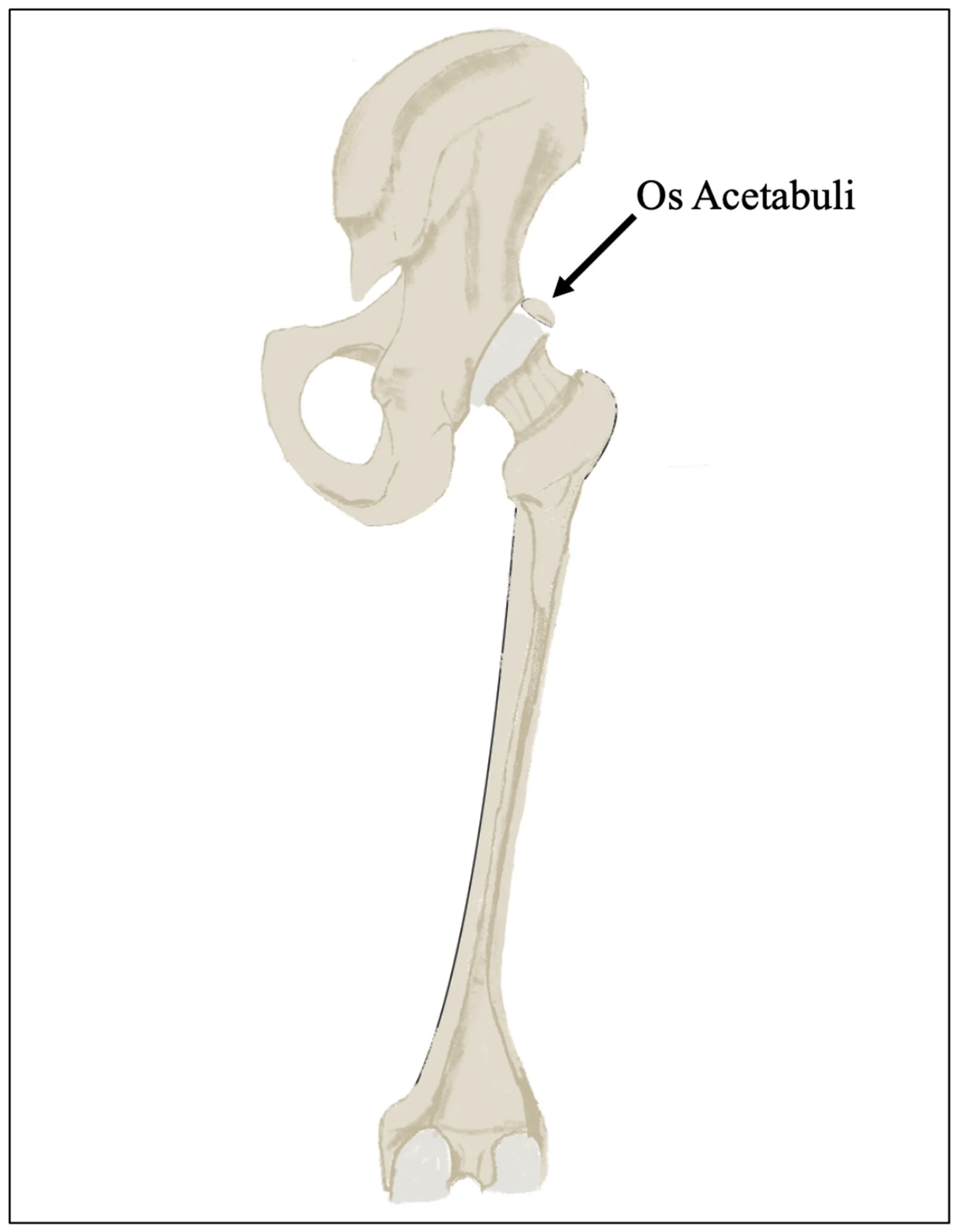
The location of the os acetabuli from a posterior view. Image Credit: Mr. Fotis Demetriou.

**Table 1:** Synthesis of anatomical variations during the posterior hip approach.

Anatomical Structure	Notable Variation/Landmark	Reported Prevalence/Evidence Base	Primary Surgical Implication
Posterior femoral cutaneous nerve (PFCN)	Mean position 30.5 mm lateral to the central tip of the ischial tuberosity [9]	Anatomical landmark; not a prevalence estimate	Potential compression or traction from distal/medial retractors; sensory symptoms are anatomically plausible [9]
Superior cluneal nerve (SCN)	Medial branch may traverse an osteofibrous tunnel near the iliac crest [11,12]	39% of medial branches in the cited anatomical data [12]	Potential entrapment or traction-related neuropathic pain; THA-specific incidence unknown [11,12]
Short external rotators	Fused piriformis-obturator internus tendon pattern [23]	10% in a 29-hip cadaveric series [23]	May blur dissection planes and complicate release or repair; outcome effect unproven [23,24]
Obturator externus (OE)	Bifid morphology with cranial and caudal heads [21]	27.8% (5/18 specimens) [21]	May obscure the distal tendon footprint; clinical consequence remains theoretical [21]
Sciatic nerve (SN)	Non-Type A SN-PM relationships; some variants traverse or straddle the PM [7,35]	13% pooled total variants; 44 studies, 8257 specimens [35]	Altered nerve course may increase vulnerability during piriformis mobilization or deep retraction [7,35,36]
Persistent sciatic vessels	Persistent sciatic artery or persistent sciatic vein [30,31,32]	PSA: approximately 0.03–0.06% [30]; PSV: rare, population prevalence not established [31,32]	Potential severe hemorrhage if a large-caliber persistent vessel is injured [29,33]
Os acetabuli	Unfused secondary ossification center along the acetabular rim [37,38]	3.4–7.7% in a review of 8836 patients [38]	Potential misidentification as fracture/degenerative fragment; relevant to rim morphology and component planning [37,38]

## Data Availability

Please contact the authors for data requests.
